# Supplementary material for: Chromothripsis during telomere crisis is independent of NHEJ, and consistent with a replicative origin
Source: Genome Res. 2019 May;29(5):737–49. doi: 10.1101/gr.240705.118 (PMC6499312; doi:10.1101/gr.240705.118)
Supplement: Supplemental Material [file supp_gr.240705.118_Supplemental_file_1.zip › contigs/annotated_contigs/DB53/contig.19.DB53_length_2532_mean_cov_21.240521327.docx]

**DB53_length_2532_mean_cov_21.240521327**

```
CTAGCAACAAGGCCGACGCCGCCTCTCTCATAACAACATAAAACC|CTAGCCGGGCATGGGGGTCCCCGCCTGTAATCTTAGCTACTCA
                                              >chr8:75097832-75098286 - E=2e-239
GGAGGCTAAGGCATGAGAATTGCTTAAACCCAGGAGGCGGAGGTTGCAGTGAGCTGAGACAGCACCCATGCACTCCAGCCTGGGTGACA

GAGTGAAACTCTGTCTCAAAAAATAAATAAATAAATAAAAATCCTTGGAAAGAGCATGAAATTAGCTGAGCATGGTCACATCCTGGTAT

GTTTATTGCTGGAGATACGAAAGAACATATTCCTACAGTTTGTACGAAGTGAAAGAAAGCCCAGCCTAGCACAAATCAGCAATCTCTGC

CAGCTCCTCAAGAATAGAAAGGAGGCCTGGGTGTTGGGTACTCAAAAAAATTCCAATGTCTACCAAAGTTTTGGCCCCATTCTACTGCA

ATTATATGGGTATTGTCCCTCTTTCTCATAAGCTCTGAACTACTCCACCCAGACA|AG|CAAATGCAGCCAAACTAAGCTTCATAAGTG
                                                          >chrX:25953670-25953715 + E=1
AAGGAGAAATAAAA|T|CCTTGCAATGGGTTCAAATATCCTCCTTTAGCTCAGAGGAGTTTGTTATTACCGACCTTCTG|A|GAGCTCT
e-11           >chr8:88806537-88806601 - E=4e-26                              >chr8:7
ATGAGCTCTTGTATTAAAAACAAAACAAACAACAACAACCCAACACATTTCTTGATCACCTATTTTGAGTCAGGCAATGTGCCATATGA
5097710-75097831 + E=2e-57
TATGGAGAATACAAAGAA|AAGCAA|ATGCTGAGAGACTTTGTCACCACCAGGCCTGCCTTACAAGAGCTCCCGAAAGAAGCACAAAAC
                   >chr8:88807300-88807655 + E=5e-195
ATAGAAAGAAACAACCAGTACCAGCCACTGGAAAAACAGGCCAAATTGTAAAGACCATCAATGCAATGAAGAAACTGCATCAATTAATG

GGCAAAATAACCAATGAACATCATAATGACAGGATCAAATTCACACATAACAATATTAACCTTAAATATAAATGGGATAAATGCCCCCA

ATTAAGAGGCACAGACTGGCAAATAGGACAAAGAGTCAAGACCCATCAGTGTGCTATATTCAGGAGACCCACCTCACATGCAAAGATGC

ACGTAAACTCAAAATAAA|G|AGCTGTCTGACCCTAAACTATCTAGTTTCTCTCAGATTACTCATTTCCAAAATGGATAATAAAATACT
                   >chr8:121100824-121101012 - E=1e-100
AAAGTGAAGGCCGGGCACGGTGGGTCACGCCTGTAATCCCAGCACTTTGGGAGGCCAAGGCGGGCGGATCGCCTGAGCTCAGGAGTTTG

AGATCACCCTAGGGAACATGGTGAAACCC|G|AGTAGAAGCATGAGAATAGTGTGACAGAAAAAGAGTGACTGCCAATCCTGACACATG
                              >chr17:68798247-68798606 + E=2e-203
ATATTGATCTTGCCATTTCCAGGGCAATTGTATATCAATCTTGCTTGGGGACCTTCTGAAAAAATGAGTAGACTTTCCTTTGACAGTGT

TTCTTAAGGGCTGGAATCTGATCTTTTGTGCCTCCAATACTCATGAGTTGCTCTATGGAACTTGTTTCCTTTCACCTTCAAGATTCTCA

TCTGTAAGAATCTCTGAGCAGTTGCCACAGGCTTCCCAGATGGTGCCCAAGAAACAGAAGGACAGAAAGGAGAAGGCAGGCAGTTCTAC

TGAGATGAGGGGCTGCCAGAGTCCACCGAGA|CCA|GTGAACACTTTATTGCAATAGGCAGCTTTCTAATCT|TCCCT|GGGAATATGC
                                >chr8:81611651-81611695 - E=4e-14      >chr8:77987710
ACGCTAGGAGAGTCCAATTTTTTGCTGTTCTACTTATGTGTGTGTCTTTGAATGACGAATCAAGTGTCACAAGCATTCATTTTTGAGTT
-77987830 - E=8e-60
ACAAACAAATTTGAG|T|TAATTGCCGATAAATGTCTTTTATGGCTCTCCAAGGTGT|AA|GTAGGCAACACAAATGTCCAAATATCAA
                >chr8:81611195-81611237 - E=6e-13       >chr8:79152476-79152565 + E=4
AAATTAGAACACTAAATGTAAATGTACTTTTTTAAGTCTGTAATCAAATGCTGAGAG|CA|TACTCACTTCCAGTCTCAGCCCTGTTCT
e-41                                                      >chr8:81610513-81610577 - E=4
CCTGTTAGCTAGAAGTACCTAGGTTGGTCCTCAT|AGAGCTTTATCAAAAAGT|GAGGAGGCACTGAGAACAAGCGAGGGCTGTGAGGG
e-26                                                 >chr8:75092256-75092323 + E=6e-25
CTGCCAGCACGCTGTCACCTCTCAATGCCA|CT|CAGAATTCTAAGCTACTTTATTTTTTATATTTATTTATTTATTTTGA|GAC|TAG
                               >chr8:75092355-75092407 - E=6e-19                >chr8
GAGGAAGGAACAACTAGTTTTGCTGGTAGAAA|CAACTCTACCACATGGTA|GAACAGTCTCTACCCTCTTCAGCTGCGGTCT|AATGG
:88456966-88457004 + E=2e-10 p=1e-02               >chr8:81610393-81610432 + E=4e-08 p
AGA|CCAATGTTATAAAAATTACCAGAGTAATGTGTTAGGGATGTGAATAAATAATAAAGATGTGTTTTTGTTTTATTTTATTTTTTTT
=3e-02 >chr8:85468702-85468829 - E=5e-64
TGTCTTTTACAAGTTCTTCATGGTCGATAGGAG|C|AGAAAAGTACTTGTTAAAATGTCACTTAAGGTTC|TAAAAT|AGCTGATAAGT
                                  >chr8:79152784-79152825 + E=3e-12  >chr17:68795569-
TAAAACTTTGATAGCAGACCCAAAAGCCTTGTCTGAACTTTGTAATACGACTGTAGTGTGTCATTATAATGTTTCATCCCTCTACTTAA
68795752 - E=1e-97
ATCGTTGCTATATTTTTCAAAAAAAGATAAAGTATAATGGATATATTTTACAATTTTCCTTAGATATTATACATTTT
```
